# May I be a sacrifice for my grandchildren—transgenerational transmission and women’s narratives of the Yezidi ferman

**DOI:** 10.1007/s10624-018-9506-9

**Published:** 2018-06-27

**Authors:** Maria Six-Hohenbalken

**Affiliations:** Institute of Social Anthropology, Austrian Academy of Sciences, Hollandstrasse 11 – 13, 1020 Vienna, Austria

**Keywords:** Genocide, Memory, Commemoration, Transgenerational transmission, Silencing

## Abstract

This paper addresses the (post)-memories of the generations of offspring of survivors of the genocidal processes in the Ottoman Empire during World War I. About 12,000 Yezidis managed to flee to Armenia and establish a diasporic community. Based on ethnographic fieldwork within this community, including interviews with members of subsequent generations, this article focuses on the narratives and experiences of women as well as gender-specific violence. The gathered empirical data makes it possible to elaborate on the hardly documented history, on its lasting effects, and on gender-specific differences in these narrations. Despite certain politics of silencing, memories of genocidal persecution were passed down from one generation to the next. The most recent case of genocidal persecution of Yezidis in Shingal (Iraq) 2014 affected the very foundations of the Yezidi community both in Armenia and the transnation—and at the same time revived their joint remembrance of the fate of their ancestors who had once sought refuge in Armenia.

## Introduction

*Ez qurbana nevîyê xwe vim*, meaning “may I be a sacrifice for my grandchildren,” was the answer an old Yezidi woman in Armenia gave to the question whether she shared her knowledge on the extreme violence her ancestors had experienced with her grandchildren.^1^ This expression underlines her strong commitment to inform her grandchildren about how their ancestors had suffered in the eastern Ottoman Empire during World War I and escaped the genocidal persecution. Although her narrations are “post-memories” (Hirsch 1997) or “vicarious” memories she had gained from her mother-in-law, she understood herself as an intermediary for transferring the fate of their ancestors to upcoming generations. While her mother-in-law survived the acts of genocide, her father-in-law was killed and five of their children died as well.

This paper addresses the (post)-memories of upcoming generations of Yezidis who survived the genocidal processes in the Ottoman Empire during World War I. It will neither contribute to the question of recognition, nor will it provide evidence in that regard. Instead, this research draws on the subject of transgenerational transmission and today’s narratives, which not only help to establish the facts of a certain event but are also fundamental in elaborating on the long-lasting effects of genocidal persecution. Although the narratives cannot serve as replacements for testimonies that were never recorded, these narrations, oral histories, or vicarious memories reflect an implicit knowledge of events that had been publicly silenced over decades. The imposed measures of silencing did not stop these memories from being upheld in family narratives—and as such remain as “deep or repressed memories,” e.g., in literary language.

In genocidal processes as well as any war against the civil population, violence against women and children has always been much more than an act of war: it is also a powerful weapon that is able to annihilate entire communities. Elisa von Joeden-Forgey (2010) stresses that genocidal violence and, above all, sexual violence, intend to harm the life force of a community by destroying its intimate relations on a family level as well as its reproductive force on a community level. Such attacks have a strong symbolic and metaphysical component with a destructive force able to conquer entire groups.

The empirical material is based on fieldwork within the Yezidi communities in Armenia. The experiences of those who survived have only been orally transmitted within families, clans, and villages—in personal conversations, in disguise, in metaphors, or in artistic expressions. As it was hardly possible for any official or semi-official narrative of these experiences to develop, the interview partners primarily renarrate the stories they experienced in their families, lineages, or village surroundings.

Interviewing female and male members of subsequent generations, the interviews are analyzed with a clear focus on the narratives and experiences of women. The main question for this paper is whether and how in the course of these persecutions gender-specific similarities and differences as well as gender violence are expressed. Furthermore, this research takes a closer look at how gender-specific experiences of extreme violence are integrated into historiography, authorized public narratives, semi-public accounts, and private (family) narratives. What kind of role did women play in the processes of transmission, as mothers, grandmothers, or mothers- in- law?

Inherent to remembering is the Janus-like character of “looking forward/looking back” (Casey 1987). In order to grasp spatio-temporal connections, the constant shaping of the past, thus the Janusian ability of remembering it is necessary to elaborate on the present developments in order to analyze the memory constructions. Therefore, I will also elaborate whether and how the most recent persecution of Yezidis in Shingal 2014 shaped the memories of genocide a century ago, affects their remembrance of the past, and revives the narratives of their ancestors’ traumatic experiences.

In a first step, I provide a brief overview of the development of Yezidi communities in Armenia and introduce theoretical approaches and considerations on the transgenerational transmission of experiences of extreme violence. This will help me to frame the contexts and most important challenges of memory work in the last decades. Then, I present relevant sections of interviews conducted with female Yezidis in the course of my fieldwork in Armenia^2^ to discuss their challenges and milestones with regard to remembrance. In a final step, my paper elaborates on the details and characteristics of intergenerational transmission, questions of temporalities, and specific female spaces and gendered expressions.

## Yezidis in Armenia

In today’s Republic of Armenia, more than 95% of the population have Armenian roots.^3^ Considering that there are several acknowledged ethnic and religious minorities in Armenia, about 35,000 Yezidis currently live in the country according to the last census.^4^ These are the descendants of those Yezidis who sought refuge from the genocidal policies of the Ottoman Empire. Most of them were from the Ottoman provinces of Kars, Van, and Iğdir.^5^ Yezidis mainly lived from animal husbandry by practicing transhumance in eastern Anatolia. They lived either in villages with other Yezidis or in multiethnic surroundings with Christian or Muslim Kurdish neighbors. In comparison to these neighboring groups who belonged to one of the three millets in the Ottoman Empire and were thus an acknowledged community with all their rights and duties, Yezidis had always experienced an outsider position and were seen as heretics, being repeatedly persecuted both by the central authorities and by their Muslim neighbors. Nevertheless, they are living together with Christian communities without violence and largely without conflict.

Unfortunately, the fate of Yezidi communities in their former settlement areas in the eastern Ottoman Empire has not been sufficiently studied yet. By the end of the nineteenth century, the Yezidis lived scattered in the eastern Ottoman provinces, the territory of today’s Turkey, Syria, and Iraq. With regard to acts of genocide in the Ottoman Empire during World War I, the international political and scholarly discourse has rarely focused on all the ethnic and religious groups concerned. As a non-Islamic denomination, the Yezidi were seen as heretics by orthodox Muslim and were persecuted just the same as their Armenian or Assyrian neighbors in World War I.

As there are no reliable figures^6^ and from the narratives told during my fieldwork, we cannot shed light on the numbers of the victims, but we learned about the planned and strategic destruction—entire villages and tribes were annihilated. The Caucasus—neighboring regions in Armenia and Georgia above all—were safe havens, attracting approximately 12,000 refugees to the territory of today’s Republic of Armenia. These refugees established a Yezidi community spread across about 40 villages and towns in Armenia (Angelova 2015). It took the survivors the entire interbellum period to develop and secure their livelihoods, and to reestablish their highly complex social, caste-like system. The Yezidi speak Kurdish dialects and are often encompassed within the Kurdish ethnicity. The main difference to Sunni Muslim Kurds is their Yezidi denomination.^7^ Other central characteristics include the oral transmission of their faith with a few written texts as well as strict endogamous marriage rules to uphold the caste-like socio-religious structure of Sheikhs, Murids, and Pirs and belonging to their religion by birth.

The Caucasian territories of Armenia and Georgia turned not only into countries of exile but also into the homes of diasporic communities.^8^ As the border between Turkey and Armenia was closed after World War I, these communities were cut off from the Yezidis in their former homeland(s)—the nation states formed after the collapse of the Ottoman Empire—Turkey, Iraq, and Syria. In the Soviet system, they were organized along the Sovkhoz and Kolkhhoz system, but were still able to continue their way of life, many of them practicing animal husbandry. They gained cultural rights, such as the operation of a radio station, a newspaper and other print media, and school instruction in their mother tongue—all of which based on the Soviet ideology of ethnicity. Due to the language spoken, thus the Kurdish dialect of Kurmanji, they had to stress their Kurdish and not their (religion-based) Yezidi roots. The gained cultural rights in the Soviet Union had always been denied to Kurds and Yezidis in Turkey, Iraq, Syria, and Iran. Armenian Yezidis, in contrast, were confronted with ambivalent, if not paradoxical, political rules and regulations, experiencing targeted ethno-linguistic promotion on the one side, and institutionalized silencing of their painful history on the other. The academic studies of Yezidis were restricted to language and folklore studies because of the Soviet ideology of ethnicity and belonging—which inhibited the documentation and acknowledgement of their horrible past from ever becoming a relevant topic. The Yezidi community could only be established in line with the reconstruction of their social system based on socio-religious affiliation. As part of the Soviet system, however, the religious aspect could not be as emphasized. This was quite challenging, as religion was a main source of their identity and yet could not be as important under the Soviet ideology of ethnicity.^9^ Due to the concept of “friendship of the nation,” violent acts of persecution in the past—of Armenians, Assyrians, Greeks, or Yezidis alike—were silenced in the Stalin era.

While the Armenian Genocide of 1915 was made a topic of concern in the Armenian Soviet Socialist Republic from the 1960s onwards, the Yezidi genocide was hardly stressed at all in the public discourse. In Alexander Hinton, Thomas La Pointe, and Douglas Irvin-Erickson’s approach (2013), the fate of the Yezidis is one of “hidden genocides,” as hardly any research has been conducted so far. Archival sources are just as rare, and the testimonies of survivors have never been recorded; only a few biographies or published family memories of influential Yezidi persons in Armenia convey the fateful history (Celîl 1982; Cindî 1999; Hecî Cewarî 2010; Îbo 2009; Şamîlov 1959).

The subsequent generations of Yezidi never forgot the persecution of their ancestors. Although there was never a common historiography or narrative, their active remembrance demonstrated both tenacity and preservation on an individual and group level. Gerald Sider and Gavin Smith analyzed historiographies of vulnerable groups in general, and state that “Plural histories emerge both against and within larger social processes—against history.” Sider and Smith examine the mechanisms involved in shaping histories, “the ways that differentiation, like silence, can be both imposed and chosen, and how differentiation, like commemoration, involves attempts from ‘above’ and below’, as it were, to claim and impose a past upon the future and upon each other” (1997: 17). In the context of this specific fateful Yezidi history, the attempts from above encompass the Soviet ideology as well as the powerful discourse on the Armenian genocide in the last decades.

Since the collapse of the Soviet Union, and due to bad socio-economic relations, Armenia has been confronted with enormous emigration rates (Rasuly-Paleczek and Six-Hohenbalken 2017). Today, some Yezidi villages are abandoned, often only remnants of once vivid communities, and of the once reestablished society and culture. Yezidis were forced to emigrate just as their Armenian neighbors. Target countries are, above all, Russia, Germany, Belgium, and France,^10^ with families scattered across various countries. My interview partners in Armenia explained that they experienced safety and security in Armenia, but the economic situation has enforced them to seek a living abroad. They stressed that nobody ever expected for such a persecution (World War I) to be repeated just a few years ago. In the last decade, however, the Yezidis in Iraq were persecuted again, in 2007 when Islamistic groups attacked Yezidi villages. In August 2014, the genocidal persecution by *daesh*, the fighters of the so-called Islamic State (IS), started in Shingal, one of the last coherent settlement areas of Yezidis in Iraq. The Yezidis were systematically assassinated and displaced, and women and children experienced the worst forms of violence and were sold like spoils of war. Yezidis in Armenia have been struck by the pictures and documentaries about the persecution of their fellow believers in Iraq since 2014. The Shingal genocide and the increasing transnationalization are key factors in the transformation of the Yezidi communities in the regions of origin and diaspora. Decisive for the latter is an increased interest in the fateful past as well as intentions for moving together and closer cooperation between the individual Yezidi communities of Iraq, Turkey (although today mainly in diaspora), Syria, and Armenia, who had experienced seclusiveness in the post-Ottoman nation states.

## Theoretical approaches—memory studies

In the last two decades, the interdisciplinary field of memory studies has evolved greatly; it has experienced a discursive explosion (Radstone and Hodgkin 2005). A number of recent anthologies and monographs have attempted to grasp the wide range of directions within memory studies (see, e.g., Argenti and Schramm 2011; Erll and Nünning 2010; Fabian 2007; Olick et al. 2011). Scholars have distinguished between “collective memory” and individual remembering, and have tackled questions of forgetting, silencing, and denial, but they stressed that every act of remembering is unique (Klein 2000: 133).

Crucial in research that has been done on extreme violence is the lifelong suffering of victims. To apply the concept of trauma in its psychological or psychoanalytical categories is confusing and little helpful to understand the formation of memory after extreme violent experiences. It neither captures nor illuminates the forces that contribute to the making and unmaking of collective memories (Kansteiner 2002: 187). Adopting the concept of individual trauma to collective trauma can be a critical issue, as researchers have to rely on empirical work (Kansteiner and Weilnböck 2010) and carefully scrutinize social facts in order to avoid the “academic fiction of cultural trauma.”

Collective trauma concepts were critically discussed and deconstructed for the purpose of studying violent and post-violent settings. Didier Fassin and Richard Rechtman (2009) or Vigdias Broch-Due and Bjorn Bertelsen (2016) have convincingly discussed the pitfalls and drawbacks of an unchallenged adoption of this concept, which has its roots in Western modernity and has become a standardized repertoire made for global consumption of trauma victims (Broch-Due and Bertelsen 2016: 2; see also Argenti-Pillen 2000).

Fassin and Rechtman, in their convincing work “The Empire of Trauma,” discussed the development of the concept and the various applications, such as “cultural trauma”, e.g., to societies which had experienced slavery, the Holocaust, and 9/11 (2009: 9), or the concept of “historical trauma,” e.g., to the colonization of Latin America and Africa or the Palestinian Intifada (2009: 15f.).

The memory of the Holocaust is, then, a paradigm for trauma, and this in two ways. First, it represents the most extreme reach of violence, and as such has become and unavoidable reference point for any experience of pain, of suffering and hence of trauma… Second, it developed after a period of silence, a fact that attests precisely to its traumatic nature. It is because of the delay between the event and its painful exposure to the public gaze that the process can be qualified as trauma. (Fassin and Rechtman: 2009: 18).

The authors acknowledge that the universalization and broad application of the concept of trauma paves the way for recognizing and going beyond the status of victims. As Broch-Due and Bertelsen (2016) explain, the trauma concept became a “universalizing force classifying and regulating the diverse lived experience of violence, suffering and commemoration,” which has “particular limits of universalizing axiomatic language and interpretative frames” (2016: 3). Fassin and Rechtman therefore argue that “The validity people are willing to accord to trauma in order to relate the experience of descendants of survivors of the Holocaust, of the Armenian or Rwandan genocide, of victims of slavery or apartheid, is not the validity of a clinical category but rather of a judgment—the judgment of history” (2009, 284). A further outline of the trauma concept would carry too far. When working with the concept, however, one has to consider the inherent danger of the continuation of victimization (Avakian 2010, 207) or pathologization (Broch-Due and Bertelsen 2016: 5) or that “the experience of trauma can be fixed and frozen in time” (Edkins 2003: 120). As research needs to keep the “*temporal dynamics* of violence (…) pertinent for certain modalities of trauma” in mind, they proposed the “notion of slow violence” to understand, e.g., the transmission and transformation of traumatic experiences to next generations (Broch-Due and Bertelsen 20`16: 5).

The transgenerational transmission of traumatic war experiences and memories of genocidal persecution became an emerging research topic in social sciences (Rosenthal 1998), and in social anthropology as Nicolas Argenti and Katharina Schramm 2011, besides others (Huber and Plassmann 2012; Leuzinger-Bohleber et al. 2017), have convincingly shown. A profound empirically based work, e.g., from Carol Kidron (2009) in her study on descendants of Holocaust survivors, shows the strength of the anthropological approach herein. Kidron convincingly shows how “nonverbal, intersubjective, embodied, and material traces of the past [exist] in everyday life, forms of knowledge that resist articulation and collective enlistment.” She also asks if “these traces subsist as ‘deep’/repressed memory below the social surface” or “lived memories” (2009:7). Veena Das argues that individual or collective experiences of extreme violence can become muted or can find expression in subtle signs and metaphoric language (Das 2007). Violent experiences are “stored” in narratives, and “archived” in language, artifacts, rituals, and bodily habits, as Regina Bendix (1996) argues, which are also in nonverbal ways handed down to the next generation as “lived memories” (Kidron 2009).

## Frames of memory work

### Historiography

After the refuge (1915–1917), the border of the northeastern Ottoman provinces was largely contested with several battlefields between the Central Powers^11^ and Russia. People suffered from several strategies of annihilation, displacements, and warfare. Once the refugees had crossed the border river Arax and reached Armenia, they were confronted with hunger and disease. In the 1918 Battle of Bash Abaran, soldiers of the Young Turkish Regime invaded (the territory of today’s Republic of) Armenia. Once again, they pursued the genocidal policy of persecuting and displacing not only the old-established population but also the Armenians and Yezidis who had sought refuge there. In the official historiography as well as Armenian narratives, the independence of Armenia was gained in the 1918 Battle of Sardarabad, when Armenian troops fought against the invading Ottoman forces. This historical account integrates the participation of 700 Yezidi warriors among the Armenian army of 5000 men into both official Armenian history and Yezidi narratives; this is reflected in memorials of these Yezidi heroes, as Christine Allison analyzed (2013a). Therefore, the heroic fighting of Yezidis could emerge as an important point in both histories, while the genocidal acts are only remembered in semi-official Armenian narratives without any official recognition. In the eyes of today’s Yezidis, this heroic male history—participation in the battle for Armenian independence—is an essential reference point in the semi-official, historical narrative of the community and its identity construction. The first memorials of these brave Yezidi fighters are found in Yerevan as well as a newly erected Yezidi cultural center. The old as well as the young generations still know various songs dedicated to the Yezidi heroes in the Battle of Sardarabad. Women are completely absent in this shared Armenian and Yezidi historiography, which focuses on nationalism and masculinized memory—men as actors and women as victims.^12^

A strategy of denial and totally silencing these atrocities was implemented by the successor state of the Ottoman Empire, Turkey, in the aftermath of the genocides during World War I—a transitional justice never took place. In line with Sider and Smith (1997), silencing is an element of power relations in memory constructions; silencing “can be both imposed and chosen.” Silencing can be a strategy of survival as well as a mechanism of control. In the presented case study, it was not the individual sphere that tried to suppress and silence the experienced brutalities. Instead, the political regime enforced this policy of silencing, which resulted in this fateful history turning into a “public secret.” In the Yezidi case, this silencing was imposed during the Stalin era, as politically undesirable individuals were displaced to *gulags* and sometimes did not return.

As Michel-Rolph Trouillot outlines based on his study about the revolution in Haiti, there are four moments of silencing: “the moment of fact creation (the making of sources); the moment of fact assembly (the making of archives); the moment of fact retrieval (the making of narratives); and the moment of retrospective significance (the making of history in the final instance).” (Trouillot 1995 in Altınay 2013, 78). In the Yezidi case, that fact that the development of narratives, even though in a semi-public sphere, could even take place in the last decades of the Soviet Union, including a few publications—the fact assembly according to Trouillot—testifies to the family history, organized as memoirs or novels (see Allison 2013b). These memories were alongside the Armenian experiences, but while a strong discourse on the Armenian genocide was elaborated, the Yezidi genocide is up till now not acknowledged officially, neither in Armenia nor in other countries with a Yezidi population. While in official national realms the historiography of genocides hardly tackled the gender issue (see, e.g., Avakian 2010), this paper shows how gender-specific genocidal violence is narrated in this long time hidden narrative scene.

### Orality

Over the decades, these memories were upheld only through Yezidi oral traditions, such as songs and oral history (Allison and Kreyenbroek 2013). Yezidi religious and historical knowledge was generally transferred orally for centuries (Kreyenbroek 1995, 2009). Children were taught in history, religious hymns and prayers, and the Yezidi’s rich poetry (Jalil and Jalil 2014, 2015, 2016). Our interview partners remembered that their ancestors had narrated their stories often like fairy tales, or that they had heard the songs of the *dengbej* (traditional singer) who expressed similar experiences in literary language. In this respect, the cultural policy was ambivalent during Soviet times. The radio program, newspapers, and academic research promoted their language, which was also essential in community-building processes. Not everything could be expressed, though, especially not their fateful past, which should have been silenced.

In the very intimate environment of their homes—when, for example, women remembered lullabies dedicated to lost family members and children—women could express their mourning and grief, and share their experiences with close friends and family members.

In general, orality is a topic inherent of the religious realm, as hardly any prayers or recitations are written. Concerning the clan history, the members are obliged to be aware of the clan or tribal history, and to recall their specific family genealogy. These expressions of orality were practiced by experts (singers) as well as the individual community members. In the Yezidi tradition, the *dengbejs* upheld and remembered historical events in their recitations, including heroic Yezidi fighters during World War I. Memories were preserved in their rich metaphoric language as long recitations. Some of these *dengbejs* became famous in the whole Armenian Yezidi community.

Women were also experienced in recalling the life of ordinary persons, especially in recitations during funerals when renarrating passages of the life of the deceased person in form of laments (Amy de la Bretéque 2008, 2012, 2013).

In other words, Yezidis are quite experienced in renarrating, in recalling long complex stories in detail. The elderly paid particular attention to strengthening these skills within the younger generation. Nevertheless, such remembrance is often fragmented, as several interview partners complained, depending on the specific situation in families or clans.

Applying Assmann’s (1992) concept of memory production, in which he argues that both reference memory (such as archives or meta-memory) and working memory (reinterpretation) are important cornerstones of memory work, may explain such obstacles. As the Yezidis were hindered to develop any form of meta-memory, thus neither an archive nor a common or public narrative could shape—the narrations were restricted to the micro-level, thus the family or village realm.

The founding of modern nation states after the collapse of the Ottoman Empire went hand in hand with the division of the Yezidis into three nation states—Iraq, Syria, and Turkey—in which they kept experiencing marginalization and exclusion. The Yezidi diasporic community in Armenia had hardly been in contact with their homeland communities for almost a century. So neither an institutionalized remembering nor a shaping of a public narrative was possible.

### Transnationalization and current persecution

In the last decades in all of these countries, Yezidis were enforced to leave due to either human rights violations, persecutions of (religious) minorities, wars, or the breakdown of the economic system. Yezidi scholars estimate that more than 30% of the Yezidis worldwide live in exile or migration and have established “new diasporas” and transnational networks in Europe (Tagay and Ortaç 2016, 31). More than 90% of the Yezidis from Turkey emigrated since the 1960s. After the collapse of the Soviet Union, and due to bad socio-economic relations, Armenia has been confronted with enormous emigration rates. So also are Yezidi villages abandoned and are often only remnants of once vivid communities. Target countries are, above all, Russia (for Armenian Yezidi), Germany, Belgium, or France,^13^ with families scattered across various countries. Nevertheless, there is also a counterflow of this tendency in Armenia originating in the transnation. In the last years, emigrants who became successful in Russia financed religious institutions, such as *ziyarets* (temples) and memorials, dedicated to the Yezidi heroes fighting in the Armenian War of Independence; just recently, a memorial dedicated to the persecutions of 1917 was erected in central Yerevan. This reflects the interest towards new commemorative and mnemonic practices for the development of a canonical remembrance (Connerton 1989, 2009). Some bottom-up initiatives, mainly in new media or social media like Facebook, have been addressing this topic and linked it to the transnational Yezidi community.

The (transnational) Yezidi community stresses its long history of persecution and argues that they had witnessed 72 *fermans*^14^*—*as the genocidal persecution is mostly called in Yezidi terms. The acts of annihilation and displacement between 1915 and 1917 were seen as the 72nd *ferman*. My interview partners in Armenia explained that nobody ever expected for such a persecution to be repeated just a few years ago. In the last decade, however, the Yezidis in Iraq were persecuted once again. In 2007, Islamistic groups attacked Yezidi villages in Iraq; they drove a lorry with ten bombs into a village and murdered 2000 people at once. This is often seen as the 73rd *ferman*. The genocidal persecution by *daesh*, the fighters of the so-called Islamic State (IS), starting in Shingal in August 2014 is understood as the 74th *ferman.* The Yezidis were systematically assassinated and displaced, and women and children experienced the worst forms of violence and were sold like spoils of war, and are still held captive to this day, their fates unknown. Shingal is still (March 2018) not safe enough for refugees to return. Yezidis in Armenia have been struck by the pictures and documentaries about the persecution of their fellow believers in Iraq since 2014.

### Multiple temporalities

In remembering the past, the Yezidis of Armenia refer to various temporalities. Against this backdrop, the theoretical considerations of Nicolas Argenti (2017) in “Presence of the Past in the Era of the Nation-State” offer a valuable approach to elaborate on the questions of chronicity and transmissions of collective memories. His intention to show the “multi-temporalities of the post-Ottoman world” based on several case studies of memory work of ethnic groups once dwelling in the Ottoman Empire is a valuable line of thinking. This approach applied to the Yezidi case provides an important aspect for taking a closer look at the manifold connections and orientations in memory work.

Dwelling in the Ottoman Empire, Yezidis were seen as “the paradigmatic heretic other” and at its best marginalized, leading various persecutions and waves of displacement. In comparison to the *ahl al-kitab*, the people of the book, thus the acknowledged religious communities organized in the millet system, their post-Ottoman perspective hardly stresses the loss of a multiethnic realm but rather the counter-history of separation and pogroms (see Argenti 2017: 7). At this point, the (symbolic) concept of the 72 *fermans* becomes crucial in today’s historical consciousness. For the Armenian Yezidis, the dispersal during World War I meant a long-lasting rupture according to a linear time concept, as they were cut off from almost all other Yezidi communities. The establishment of their community in Armenia within the Soviet sphere, influence, and ideology brought forth an existence in a completely new environment characterized by safety and the promotion of cultural and linguistic traditions yet also by the silencing of their past and their main source of identity, the religious denomination. Instead, they were adjusted to new common belongings in space and time. Establishing their lifeworld in the post-Soviet nation state, the enforcement of migration was expanded and increased their transnationalization but also allowed a new rapprochement to all other Yezidi communities. Today’s transnational community is shaped by various transformations, such as the intention to elaborate, on the scriptualization/textualization of their religion based on orality and orthopraxy (see Omarkhali 2017) or on a common historiography in transgressing boundaries—a result of dwelling in different nation states. The most recent persecutions in Shingal/Iraq in 2014 imposed various challenges on the Yezidis in Armenia and tackled different conceptions of temporality. The Yezidis in Armenia explained that they would never have expected an act of persecution as the one their ancestors had experienced to ever be repeated. In their eyes, Shingal 2014 and World War I are just like two mirrors, constantly reflecting the image of the other, the presence in the past and the past in the present. In order to be able to grasp the extreme violence, the loss of one of the last coherent Yezidi territories, and the current state of insecurity in Iraq, Shingal is seen as the 74th *ferman* in the Yezidi traditional worldview.

Referring to Argenti’s critique on the “unilinear model of historical time” (2017: 13) as a Western construct, the narratives hardly follow unilinear time concepts in this Yezidi case study. The memories of the people relate to “multiple registers of time: linear and cyclical, modernist and anachronistic, empiricist and subjective/affective, collective and individual, deep and diurnal” (2017, 13). The following presentation of empirical material reveals various time concepts. As mentioned above, the loops of temporality have no gender-specific characteristics but are important for analyzing the multilayered narratives of women’s accounts and female suffering.

## Narrations—gendered memories

Accessing the often remote Yezidi villages of Armenia with my research collaborator—herself a young female Yezidi academic—we asked the villagers if they remembered what their ancestors has passed on to them. Most of the villagers as well as those Yezidis staying in urban settings were willing to share their family stories with us.^15^ We sat down with them and listened to the witness accounts they had heard from the survivors. Many of them started to recall their family’s and village’s narratives right on the spot without ever taking a break. These narratives belonged to them; they could recall them without hesitation.

G.^16^ belongs to a Pir family, which means that she is from the clergy caste and in her 70s. She lives near the Turkish border in an extremely remote village that has been almost entirely abandoned as a result of massive emigration rates all over Armenia. In her narrations, she focuses on her father’s family, how her grandmother who had five sons managed to escape with three children, and how one child died on the run.

*(…) our ancestors, my ancestors were just from the village of Armenians. My grandmother sought refuge with Armenians. The Armenians protected her and warned her, saying “take your three children and run with us because if you remain, they will come and kill you. Run with us, they [two sons and other family members] will save themselves.”*

G.’s grandmother knew that her husband had been killed, but she did not have any information on the fate of her two elder sons who did not manage to flee with her. She told us in great detail what happened to her grandmother, ranging from the various perpetrators to the different cruelties committed against men and women. The acts of violence against women ranged from mass killings—even pregnant women—to sexual violence, when she said that “they went to the women by force” and humiliation, when the perpetrators cut the women’s long hair and plaits as trophies. Women had hardly any agency in defending themselves, as they were not used to fighting with weapons, and they could only try to protect younger women by dressing them up as elders. G stressed the long period of suffering and hardship during the post-genocide period. Her grandmother went begging at the Yerevan train station to feed the two sons and protect them from orphanage, while always waiting for her two other sons. First, there were rumors that they had been killed, but later she learned that they had survived in Turkey. As the border was tightly closed after the war, she had never seen them again.

I recorded G.’s story twice, and in both instances, she moved her body to show how her grandmother had to use her bare hands to dig a grave for the deceased child while on the run.

G. recalled the situations when her grandmother told her about the fate of their family members, well aware of how intimidating these narrations could be for the children:

*She [the Grandmother] was able to speak about it; she gathered us around a little oven and she said to us, “Children do not be afraid, there is no war here, don’t be afraid. Do you understand?” She encouraged us, she encouraged us, and my father also encouraged us. They said, do not be afraid, the cerd,* 17 *the Rom [Ottoman soldiers] would not come here anymore.*

It was important in G.’s outlines to emphasize, that her grandmother “was able to speak about it,” that she found words, that she was willing and able to tell her experiences to her grandchildren, and that she was aware of the intimidating and traumatizing contents of the family story.

Where are the similarities and differences between G.’s narrations and those of others? Compared to the memories of several other women, especially lay people, thus from the Murid caste, the women often retold only stories of the husband’s family, where the mothers-in-law were the main sources of transmitting both family and female experiences. G. belonging to the Pir caste, was one of those who told us both sides.

There has never been a ritualized or common commemoration; there were neither specific spaces or places or a defined time. Remembrance took place “occasionally” during the everyday work or in the informal day-to-day meetings. There is no definite structure, time, or place of intergenerational transmission apparent. In some villages, there were specific places where men gathered, spent their spare time, and also retold their family experiences, but where women were almost excluded from these places. In other villages, there were specific spaces where the elder (female and male) survivors gathered and shared their grievance and their stories. Again, in other villages, people had specific spaces or times when they would remember their fate—e.g., when elders met each other in the village, or gathered the children around them and told them about the persecution, sometimes like fairy tales. When these witness accounts were just transmitted within the household, the stories of the mother-in-law or grandmother were the main source for my female interview partners.

Elder women often remembered the horrible deeds of the past during specific everyday work, as G. explained:

*Grandmother Seyro, her name was Seyro, used to sing songs about the children, her children who remained there (…) yes, yes, she used to tell lullabies. If she did not tell lullabies, the children would not sleep. (…) We had a cow and she used to milk that cow and when she sat to milk that cow, she told the songs about her children, even when milking the cow. She used to cry when milking the cow and she told us about it.*

Several women and men retold details of their ancestors’ escape similar to G’s story—e.g., how women with tiny children or children alone escaped, how women heroically saved their children, or sometimes how they could not manage to save all of them. The agency of women was acknowledged, but they also stressed how limited their scope was.

J., an old woman living alone in a border village to Turkey, recalled the fate of her family. She was never married and stayed in the paternal household. Her parents did not experience the genocidal persecution as several others did, but she explained that at the end of World War I, when the border was once again contested, her ancestors were expelled from their village on the Ottoman side of the border. They were driven out, forced to cross the border river Arax, and settled on the other side of the frontier.

*Yes, they passed Arax River (…). If we had the right to go to the river, I could show you. There is a place, actually the river is deep, but there is a place where the river is narrow and it is possible to cross it easily, but there is the fence and it is not permitted to go. It is not far from our village.*

So her parents had not experienced the strategies of annihilation, but instead those of expulsion as well as the loss of their homes and villages, which were in visual range from the village in Armenia they were living in, trying to cut out a new existence. Today’s memories of that time are closely connected to the landscape, as villagers still recall the battle sites and incidents in their vicinity, as if it had happened just yesterday. They pointed to certain mountain ranges, gorges, or, in this example, a specific place at the border river, where people managed or did not manage to cross.

Several interlocutors expressed that their ancestors were longing for a return. One striking characteristic of the interviews was the fact that several ancestors had never been able to find out what had happened to their family members. While some stressed that their ancestors witnessed the mass executions and that they were the only survivors of a big family, village, or lineage, the whereabouts of the family members of others remained unclear. So silencing perpetuated the uncertainty.^18^ The silencing of the atrocities in the Soviet Union lasted several decades until a new deployment in Armenia became possible. Detailed documentation started from the 1960s onwards, when several state archives opened their collection of diplomatic correspondence (Adalian 2009: 75). Yet, not before 1965, a commemoration of the mass atrocities was possible in the Soviet Regime (Tölölyan 2001). A few interview partners in Armenian frontier villages argued that their parents were aware that certain family members had remained in Turkey. They only rarely received any information, but most of the time it was impossible anyway. The border between Turkey and Armenia was tightly closed for decades, and there are still no border crossings to this day. Only in the last decade has it become easier for Armenian citizens to travel to Turkey via Georgia. Some elderly Yezidi men decided to travel to Turkey to find their parents’ villages and homes, to fulfill the parents’ dreams, and hoped to find hints about missing family members. Only in a few rare cases did they learn anything about the fate of relatives and their descendants. It is such cases of uncertainty, however, that fuel the remembrance process as well as the Yezidis’ imagination of existing relatives across the border. Uncertainty of their relatives’ fate kept the families in an interim state upholding hopes and memories. Even if the memories were fragmented or silenced in public, however, this uncertainty was an additional factor in upholding the remembrance as “post-memory” among upcoming generations. Only in recent years, there have been attempts to develop sustainable relations with the wider Yezidi (transnational) community and to look out for descendants of missing relatives.

These both stories hardly differ from male narratives with regard to their structure, the way of narrating, their intentions of being precise, and to topics remembered and expressed. Both have in common that they are based on or include the fate of their family of origin or on the in-law’s history.

In a remote village, a woman in her 60s shared with us the story of her father-in-law and a paternal aunt. B. said:

*Yes, he [father in law] was a child, he was approximately five years old. His [paternal] aunt, her name was Sheni, saved him. Now all of them are dead. (...) Sheni crossed the Arax. She said when they escaped, they just managed to take their cart, and the cart was a cart of bulls or oxen, and they put on that cart their little millstone and a sel [a sheet of metal for baking bread]. Sheni said: “We saw a bride with her veil and she was thrown into the river; she was trying to come out of the water, and the veil was on the water, and the water was very powerful and took the bride. The bride was thrown in that water and she tried to come out. But nobody could catch her and we crossed the river with the carts.”*

Arax River was the natural border between the Ottoman and the Russian Empire, and is still today between the successor states of Turkey and Armenia. Even though this border was sometimes contested especially at the end of World War I, managing to cross the Arax, which was dangerous in itself, meant reaching save territory. The Arax is sometimes also called “Black Water” to express the tragedies, sorrows, and traumatic memories which are connected to the river. The informant stressed that only two of her in-laws had survived—the father-in-law and his aunt—who has become the source of her knowledge. She expressed that they had only been able to take whatever they needed to make bread but had lost everything else. The second passage in this narrative about the bride who drowned in the river is closely connected to the attack of the life and reproductive force of the Yezidi community. In some interviews with similar highly symbolic narratives, the bridal couple was not only a symbol for the continuation of biological existence but also included a sacred element. Yezidis indicated that in battles, for example, a bridal couple was spared from persecution, as it would be against a sacred principle and offer an opportunity of showing mercy. In other words, the acts of persecution were not only inhuman but against the divine order. The failed attempt of the community to help the bride out of the water is connected to the irretrievable loss of a part of the Yezidi community due to the persecution.

This shortened version of the narrative expresses the almost total annihilation of the community: only a few of the community were saved—two out of a huge family survived—they could secure only the mere survival—the bread—while the reproductive force of the community was assaulted—the drowning of the bride. In this condensed form, she expressed the trauma the Yezidis experienced. “The ensuing trauma of witnessing a whole life world unravelling, sometimes built over centuries, is a collective form of suffering which remains lodged deep in memory over generations” (Broch-Due and Bertelsen 2016: 6).

Ayşe Gül Altınay criticizes that in genocide historiography women and children are often treated as an entity, being under a male protection and without own agency (2013: 88). In the female Yezidi narratives, although one can trace this categorization “woman and children,” women are active agents and subjects.

S., a woman in her late 60s, recalled the story of her mother-in-law, and specifically stressed that the community forced her to leave her small child and seek refuge. The fleeing population was sometimes forced to hide in the woods or in caves, until they would see an opportunity to proceed. Therefore, babies were seen as a threat to the community because their screaming could reveal their hiding places. S.’s mother-in-law was against the decision to leave her baby; she returned and saved her child. Sometimes criticism was voiced against gender-specific differences—leading to forms of violence—within the community, when, for example, some families tried to save their boys when crossing Arax River and left the girls behind or let them drown. Such narratives express the inconceivable violence they experienced, which evoked a violent behavior among survivors in order to struggle for their life. Such acts destroy the intimate relations within a family, and the trust between parents and children and between couples, and evoke self-reproaches and self-doubt for having survived. These forms of evoked physical violence against members of the own community, either against women or against the weakest parts of the community, stands in ambivalence to the example of the bride who drowned in the river Arax. The female body was under several constraints, ranging from physical annihilation and symbolic destruction to sexual violence harming the individual and the reproductive force of a community, to acts of violence from the own community in the struggle for survival, in which males were at an advantage in extreme situations.

Despite the impossibility to document and write down this fateful part of the Yezidi history, and despite the silencing under the Soviet regime, people were well-informed about the various strategies of annihilation, waves of refuge, the displacements, the subsequent violence, and the struggle for survival after their refuge. As the Yezidis are a community with a strong focus on orality both in religion and in social life, the narratives were accurate, detailed, and precise (see below). Almost all our interview partners remembered the place of origin of their ancestors, coming from villages of the bordering provinces Kars or Igdir/Iğdır,^19^ or fewer people from the far-off provinces of Van or even Mosul. Moreover, both men and women clearly outlined the reasons for the ancestors’ refuge, ranging from brutal attacks to kill everybody in the region to abduction, displacement, warfare actions at the Ottoman-Russian border, or fleeing twice, when people tried to resettle in their villages and were again persecuted. They often recalled the names of the family members who were assassinated, knew the specific circumstances of how one or another person could have been saved, and stressed the suffering as well as the various places of settlement in the aftermath. Some had wide-ranging knowledge of the economic conditions their ancestors lived in before the persecution and knew a lot about material loss.^20^

X., a woman in her late 70s, belongs to a Sheikh family, the highest religious caste. As in the case of G. from the clergy Pir caste, X. recalled stories of both the father’s and the mother’s side, and also stressed that women had to defend themselves with weapons. This is a form of agency of women only a few interview partners mentioned. Both directly and indirectly criticized the privileged position of Yezidi men. In the post-genocide setting, this meant saving the male offspring first and caring better for them. She was taught that “girls belong to others,” which means to the future husband’s family.

*My father and my mother were from Kars. My mother herself was originally from Kars. My mother-in-law was from Van. (…) My mother said, “My brother was a little child, (…) our parents died. My uncle’s family took care of us [when they were fleeing]. I hang the holy relic around one side and I took my brother on the other side. (…) We crossed the river and one of the wives of one of my uncles was pregnant and was going to give birth to the child.”*

In the previous passage, X. also mentions the rescue of holy relics, which in some narrations was as precious as saving a child. As there are hardly any institutions, such as churches or mosques, holy relics are preserved in the houses of Sheikhs and Pirs, running centers of religion and pilgrimage, so-called *ocaks.*^21^ Holy relics are until today stored in the Sheikhs or Pirs houses, stored and shown publicly only on very specific occasions. Saving a cult object at the time of genocide was recounted as a miracle and, of course, was a vital prerequisite for rebuilding the community. There were quite a few accounts about how ritual objects were saved, hidden on the back of young girls, which has a double significance—saving the force of spiritual reestablishment through these objects and the physical reproduction of the community through the female body—as it was stressed in X.’s narrative, when her mother managed to save both. This double saving of the community’s life force was confirmed and strengthened in the short passage on an aunt giving birth while on the run. Just after having crossed the border river and reaching safety, this clergy family experienced loss but also the reproduction of their community.

In general, in all the narratives, the fates of women were not silenced; they were mentioned as victims and survivors, experiencing the overall annihilation and witnessing gender-specific violence. Incidents where persons heroically saved family members or villagers, regardless of their gender, were remembered likewise.

Women and men unsolicited told us about gender-specific violence, but they always expressed it in general and never related it to a specific person or family. Violence against girls and women was mentioned in the narrations of both male and female interview partners, although it was burdensome for them to express. Sexual violence against Yezidi women meant annihilating the community in its core. As Allison Ruby Reid-Cunningham argues, sexual violence “is often a cornerstone of genocidal campaigns because of its devastating effects on women, families, and communities (…) causing serious bodily or mental harm, deliberately inflicting conditions of life calculated to bring about the group’s physical destruction,” (2008: 280). In 1918, the invading Ottoman soldiers occupied the territory and continued a policy of annihilation and displacement. As a war strategy, women in various villages were systematically kidnapped, taken hostage in military camps, and only sent back to their villages after days of sexual abuse. Although the narrations are connected to specific locations, as people often pointed to specific places in the landscape where the aggressors had stayed, they usually stop afterwards without further outlining what happened to the women who—in the Yezidi concept of honor and shame—had been dishonored.

## Specifics of memory construction

### Intergenerational transmission and emotional remembering

Several interview partners were confronted with their ancestors’ experiences and had heard their stories at a young age, starting approximately when they were 8 to 10 years old. One Pir woman said that she could not grasp everything but was aware of their ancestors’ pain and suffering. One informant strikingly said, “They killed themselves when they remembered.” Others retold specific situations when their ancestors had been lost in their memories and grief during every day practices. If such experiences were recalled in group settings, they were always informal and not official, as people were afraid of talking about these topics, primarily during the Stalin era. One woman whose father has been exiled and died in a gulag said, “Did the Bolshevik [system] let us see our father, listen to his words?” This was not some kind of a rhetorical question, but instead means that the political system made even the transmission within families impossible. This was another factor how the development of official forms of remembrance was hampered, enhanced by the fact that families of leading religious clans as well as heroic fighters in the Battle of Bash Abaran—fearing their influence and power in the Yezidi community—were often exiled to gulags for several years. As this policy of silencing^22^ was implemented in the Stalin era and beyond, there was no way for official narratives, a historiography, or any culture of (semi)official remembrance (memorials, days of remembrance, rituals) to take shape, but these narratives remained as “public secrets” (Taussig 1999) in the oral history of the Yezidis.

Several of the elderly interview partners complained about their own truncated memories, as they could not recall all the narratives they had heard from the ancestors. They were aware of the hardship and suffering and could still recall specific situations where their (grand)parents were lost in their traumatic memories. In other words, the younger generation was brought up with a specific “emotional remembering,” encompassing their ancestors’ inability to verbalize all their fates, while experiencing their mourning and suffering.^23^ In *Remembering the Holocaust: A Debate* (2009), Jeffery Alexander stated that the narration and memory of mass murder can only be grasped after two or three decades. The first reaction of victims is to be silent about their experiences, since words cannot describe such “ontological evils” (Eli Wiesel). Veena Das also stressed the impossibility to narrate (2007).

“Emotional remembering” is a topic outlined by Bloch in his fieldwork among the Zafimaniry of Madagascar. He discussed vicarious memories—i.e., transmitted memories—of events before the interview partners were born. “The children (...) were imagining the past, but this past was attached to direct emotional and empirical experiences of the same type as if we had been there. What was being stored in our mind was therefore a mental model containing both imagery and emotion (...).” (Bloch 1998, 120). Similarly, in specific unofficial settings behind closed doors, the traumatic experiences of the Yezidis were passed on, while the silencing in the public realm was continued at the same time. The subsequent generation was not only fully aware of the fateful history of their family or clan, however, but grew up with strong emotional connections to their ancestors’ fates and the imposed silence. In the interview situation itself, several elderly people were emotionally touched when retelling their (grand)parents’ experiences. The silencing and inability to publicly express, reenact, and memorialize the acts of genocide was a result of the confluence of various factors, directed by Soviet policies against minorities, power factors in the region, and the political ideology in which socio-religious origins and belongings could not be addressed.

Some elderly men emphasized that they had gained their knowledge from what was told in the “men’s spaces” in the village, in the *ode* (the village guestroom), or in places were elderly men met. Some reflected on the gendered spaces in the villages, the guesthouse, the household, or the *tandur* (traditional oven for baking bread), where they had learned of the fates of their ancestors and villagers, while others mentioned groups of elderly men and women meeting, sitting together, and mourning together. Women often explained that they remembered their grandmothers mourning during specific daily works, like preparing special dishes, which reminded the mothers of their deceased children.

Women are undoubtedly a key factor in the processes of transmission. In comparison to the male narratives, the women’s stories are more concentrated on the family and single family members, while male narratives often enclose the clan or tribal history. In the memories recorded, there is a certain tendency suggesting that the stories of survivors were transmitted gender-homogenously as well as in patrilineage. Although several interview partners of both genders explained the fates of their fathers’ and mothers’ families, the women’s narratives were predominantly transferred via mothers-in-law. In consequence of the strict rules of endogamous marriages (within the religious caste), the rather low marriage age, specifically of girls, was also intended to prevent any sexual intercourse before marriage. In general, the young couple was obliged to stay in the paternal household, so women rather identified with the narratives of their mothers-in-law or grandmothers than with their own families. Perhaps they were simply too young to grasp and remember the narrations their mothers and grandmothers had told them and therefore recalled just the stories of the husband’s family.

Elderly people sometimes follow one of their children to Europe or Russia. Several interview partners were desperate and argued that the memories seemed to be lost, as the chain of the intra-familiar transmission was often not intact anymore.

### The past in the present—the present in the past

I started my field research 1 year after the acts of persecution in Shingal. Several informants argued that today people would speak much more about the *ferman* a century ago. Their raised awareness, the persecution of all the Yezidis in Shingal, and the representation of gender-specific violence in the media might have influenced and shaped their narratives. This development also led to the demand for international acknowledgement of the genocide a century earlier.

After having retold the fate of their ancestors, the genocidal persecutions of Yezidis in Shingal (Iraq) since 2014 played a decisive part in the discursive segment of the interviews. Almost everybody referred to them: the mass executions, the displacement of the entire Yezidi population from Shingal, the abduction of women and children, the dwelling of survivors in refugee camps in the Middle East, the precarious situation, and their unsecure future have had dire consequences on the transnational Yezidi community worldwide, and thus also on the Yezidis in Armenia. Almost all the Yezidis in Armenia had followed the media coverage on television or online, which revived the memories and fates of their ancestors.

In the course of my field research, recounting violence against women did not seem to be too shameful or dishonoring (for the person recounting it) to be silenced, denied, or tabooed. Against this backdrop, the question of the present in the past, and in the Yezidi case, probably the media coverage of genocidal processes of the so-called Islamic State against Yezidis in Shingal in 2014, where gender-specific violence was always discussed on the global level, might prove to have an influence on today’s narratives.

In witnessing the crimes on television and in new media, mass executions which the IS publicly performed and transmitted on their own TV channel were broadcast by other Middle Eastern TV stations and in social media. The visual material of these crimes reminded them of what their ancestors had experienced and narrated. Several believed that history was about to repeat itself and drew parallels between the persecution of Yezidis today and at the time of their parents and grandparents. In other words, their post-memories are coinciding with the documentation of persecutions, with the testimonies of abducted girls, and the situation of the survivors in refugee camps today. In Argenti’s approach (2017), the multiple temporalities co-exist—the past *ferman* during World War I remains present—and when witnessing recent acts of persecution in Shingal, the traumatizing memories appear to return. The media coverage on the fate of Yezidi women and children who were sold like slaves raised international attention. It also appears as if this awareness fostered narratives on gender-specific past violence.

## Women’s expression and hidden scripts

Official forms of commemoration were forbidden over decades, but Sheikhs in Armenia remember the victims of the genocidal persecution during the prayers until today. The role of women in the funeral rites are decisive in ritualized mourning and lamentations. The ethnomusicologist Estelle Amy de la Bretèque recorded and analyzed the tradition of lamentations in Armenian Yezidi society. These lamentations are a melodized speech, called *kilamê ser*. The improvised recitations are solely performed by women mostly at funerals or graveyard feasts (*roja mezela*) and may also be inserted into daily conversations when the topic evokes sad memories (Amy de la Bretèque 2012: 132). These expressions of sad feelings are practiced at funerals, when female specialists express their mourning and direct their words either towards the person deceased or towards the audience to refer to the life story of the dead person.

When the female mourners assemble around the dead body in the house of the deceased, they recite *kilamê ser* almost all day long until the person is buried and contrast the short prayers and holy songs (*qewl* and *beyt*) recited by the clergymen during the inhumation. In these recitations, the exile *xerib* is a topic either when people died in migration or when women tackle the refuge 1915–1918 or in general lament about the brides moving to the paternal household (Amy de la Bretèque 2008: 63f.).

Christine Allison studied the Yezidi oral traditions in Iraqi Kurdistan in the 1990s and also analyzed the lamentations recited during funerals (2001: 167ff.). As in Armenia, lamentations are solely practiced by the women and are performed within the 40 days of mourning (2001: 178).

Although an institutionalized commemoration was impossible, women practiced ritualized forms of remembrance, where they were the acknowledged and important actors. While in the Armenian Yezidi realm women hardly acted as *dengbej* (singers/reciters of the oral history in nonreligious settings^24^), their role in the realm of religious practice during funerals was essential.

For both the *dengbejs* and the female reciters of *kilamê ser*, the rich Yezidi oral literature and literary language allowed for alternative expressions and “hidden scripts.” The expression “kok kalandin,” for example, meaning that “the roots were fried,” is referring to when a tribe lost its roots and was annihilated. Another expression of such kind was used when they spoke about the “country which had a ‘*gazo*’”—*gazo* means nectar or the Biblical *manna*, so the lost homeland was seen like a holy land providing them with *gazo*.

Specifically, when the persecution was silenced (Stalin era) but were a public secret,^25^ alternative expressions or a language of defacement (Taussig 1999) were used which did not fully expose the speakers but were forms of hidden scripts conceivable for the audience.

## Conclusion

Although the development of any official narrative was impossible in the case of the Yezidis, violent experiences and especially violence against women were alternatively expressed in metaphoric language, in everyday settings, and sometimes like fairy tales. Only in the last few decades has the heroic (male) Yezidi history in the Armenian War of Independence transgressed Yezidi oral history to become an official aspect of remembrance (memorials, commemoration).

Against this backdrop, the memories were transmitted to upcoming generations both willingly and unwillingly, and were preserved in oral history and literature, often in hidden transcripts or family narratives. In the families, the women had an important role in transmitting the family history—in lullabies or stories like fairy tales. The various forms of transmission among elderly people full of mourning and grievance resulted in the children growing up with the need to cope with the memories of their elders. These are not memories connected to a factual history or a linear history but closely connected to emotional expressions of traumatic experiences as well as feelings of loss and uncertainty. They show the relatedness and co-existence of multiple temporalities in which specific incidents of the past remain present. Although being silenced, alternative expressions and a literary language, or a language of defacement, allowed the integration in funeral rituals.

The fieldwork for this paper was undertaken in 2015 and 2016. People then were seriously affected by the fate of the Yezidis in Shingal. They followed the media coverage on TV and in social media. The visual documentation of the persecution overlapped with the narratives they experienced from their parents or reevoked long-forgotten memories.

Armenian Yezidis are aware of the lasting effects of persecution, and of the challenges for the next generations. Sometimes, they could hardly verbalize all they had had to cope with, but their emotional connection to these issues was always more than apparent. They had to grow up with parents and parents-in-law who struggled with their horrible experiences and suffering. Male and female stories of survival were of the same importance and were retold in various ways.

These memories have been inscribed in the everyday lives and self-understanding of the witnesses, so the subsequent generations can still recall everyday situations, when their relatives remembered or recalled their fates. The violent experiences “archived” in oral history, in family histories, in a metaphoric language, and in social practices intermingle with the current persecution of Yezidis in Iraq. In consequence, their emotional remembering has been reinforced, just as the ways and intensity of speaking about their ancestors’ fates. Their post-memories are interlinked with the current suffering of Yezidis in Iraq, in which gender-specific violence is imminent.

## Footnotes

1 This research was enabled and funded by the FWF (Fonds für Wissenschaftliche Forschung/Austrian Science Funds, Elise Richter Programm) and the Institute for Social Anthropology (AAS). I am greatly indebted to Dr. Tereza Amryan, whose comprehensive support and advice made this field research possible, and the two reviewers for their valuable comments on a previous version of the paper.

2 My outlines are based on fieldwork in the Republic of Armenia in 2015 and 2016, including about 80 narrative interviews, a questionnaire, and participant observation.

3 Acknowledged ethnic and religious minorities are the Yezidi, the Assyrians, Greeks, Jews, Ukrainians, or Russians (Asatryan and Arakelova 2002).

4 According to National Statistical Service of the Republic of Armenia (2013), a total of 35,308 Yezidi lived in Armenia in 2011. Compared with earlier statistical surveys, this number indicates high rates of emigration, as some 40,620 Yezidi lived in the country in 2001.

5 According to official statistics, about 324 Yezidi were counted in the territory of Armenia, mostly in border villages to Turkey, in 1830. As this number had increased to 12,237 individuals by 1926, some 12,000 people managed to flee from the provinces of the Ottoman Empire (Mkrtumyan 2000: 28–32)

6 Estimations speak of up to 200,000 people. http://www.aina.org/news/20080307153331.pdf. Accessed 22 May 2014.

7 A monotheist religion with strong interconnections to Zoroastrianism, Christianity, Judaism, and Sufism (see Guest 1987; Kreyenbroek 1995)

8 In the Soviet Republic of Armenia, this diasporic community of Yezidis spread across about 40 villages exclusively inhabited by Yezidis, further villages where they lived side by side with their Armenian neighbors, and Yezidis residing in urban centers.

9 For many Yezidis, a self-definition as Kurdish was always interconnected to Sunni Islam. The construction of inter-/intra-ethnic boundaries as well as the question of belonging to and being a Yezidi or Kurd presented a major challenge that only began shifting after the Soviet period (see Dalalyan 2012).

10 Comparing the census of 2001 and 2011, the Yezidi population decreased by about 14% in just one decade. This development, caused by emigration, already started in the late 1980s and is still continuing today (Schulze and Schulze 2016: 378).

11 The German Empire, the Austro-Hungarian Empire, and the Ottoman Empire.

12 See also the general discussion of gendered national memories in Altınay 2013 in reference to Cynthia Enloe.

13 Comparing the census of 2001 and 2011, the Yezidi population decreased by about 14% in just one decade. This development, caused by emigration, already started in the late 1980s and is still continuing today (Schulze and Schulze 2016, 378).

14 The number 72 also has a metaphorical meaning in prayers and oral history.

15 The interviews were organized in various social groups (urban and rural, uneducated and educated) and religious castes (lay or religious), conducted and audio-recorded in the specific Yezidi dialect of Kurmanji.

16 All names of our interview partners are anonymized, with the initials just serving as synonyms.

17 I thank Dr. Tereza Amryan for this linguistic explanation. In the language of the Yezidi, there is no term specifically for genocide. In the interviews, people used komkujî (mass murder), cerd, or ferman. The term cerd comes from the Armenian language and means “to break” and in a figurative sense “to massacre, to annihilate.” Ferman comes from Persian and means “decree.” Here it refers to the several decrees in the Ottoman Empire, which legitimized the violence against the Yezidis from the sixteenth century onwards. “Ferman” or “cerd” is a kind of “internal language” signifying and constructing the past (see also Zerrin Özlem Biner 2010 referring to Veena Das)

18 This state imposed that silencing differs from the chosen forms of silencing as Kidron has shown. Silence and the loss of words must not only be seen as a “psychologically maladaptive repression of the past, the failure of interpersonal interaction, or ultimate forgetting and absence” (Kidron 2009: 16).

19 In Turkish Iğdır, the Armenians and the Yezidis spell it Igdir.

20 Similar oral history studies have shown that in comparison to spaces where the genocide was not silenced but denied, as in Turkey, the remembrance is less detailed. In Biner’s study, people avoided to mention names, places, or dates (2010: 80)

21 These are seen as holy places, where people come to show their devotion and ask for relief. In some Sheikh families, specific healing practices are known. Certain families have special skills in healing specific illnesses (e.g., childhood illnesses, psychological problems), and some of these Sheikh families were known even beyond ethno-religious borders. This knowledge is mostly kept and only transferred within the family regardless of gender.

22 For the policy of silencing, see for example Green 1999.

23 See also the concept of “emotional inheritances” (“Gefühlserbschaft”) outlined by Angela Moré (2015).

24 See Marlene Schäfers convincing study on memory, oral history, and the contribution of (Muslim) Kurdish female *dengbej* in Turkey (2017) and the publication of Hamelink (2016) on memories and traditions of oral recitation of *dengbej* in Turkey.

25 A known evidence or historic facts which *cannot be spoken*; see Taussig 1999.
